# Dimensions of Trust in Health Care Experiences Among Latino Patients: A Scoping Review of the Literature

**DOI:** 10.1007/s11606-026-10591-4

**Published:** 2026-07-07

**Authors:** Frania Mendoza Lua, Ka’Derricka M. Davis, Andrea Banuelos Mota, Evelyn Luner, Mary Hitchcock, Maria Mora Pinzon, Yohualli B. Anaya

**Affiliations:** 1 Department of Population Health Sciences, University of Wisconsin-Madison School of Medicine and Public Health, Madison, WI USA; 2 University of Wisconsin School of Medicine and Public Health, Madison, WI USA; 3 Southern California Permanente Medical Group, Mission Hills, CA USA; 4 Department of Family of Medicine and Community Health, University of Wisconsin-Madison School of Medicine and Public Health, Madison, WI USA; 5 Ebling Library, University of Wisconsin-Madison School of Medicine and Public Health, Madison, WI USA; 6 Department of Medicine, University of Wisconsin-Madison School of Medicine and Public Health, Madison, WI USA

**Keywords:** trust, communication, patient-centered care, primary health care, health care disparities, special population, underserved/minority, scoping review

## Abstract

**Background:**

Trust is critical for improved management of chronic conditions among minoritized communities, whose history of exclusion may affect their ability to trust health care institutions. Among these groups, Latinos report the lowest levels of trust, making it essential to understand what fosters trust in the health care system. This study is a systematic examination of the literature to identify and map data on Latino adults’ trust in health care of chronic conditions.

**Methods:**

This scoping review involved a comprehensive search of four databases, PubMed, Scopus (Elsevier), SocINDEX (EBSCO), and PsycINFO (EBSCO), for peer-reviewed articles on health care–related trust among Latino adults and chronic disease care utilization in U.S. primary care between 2004 and 2024. Screening and data extraction took place in Covidence. Two researchers reviewed full-text articles independently. Eligible studies were mapped onto the Health Systems Trust Content Area Framework and analyzed using deductive content analysis to provide a representation of the data and new insights.

**Results:**

Twenty-eight articles met inclusion criteria. Most studies focused on communication and global trust. Dimensions expanded existing domain definitions relevant to Latino patients: language access, provider-patient concordance, and cumulative health care experiences. Stigma that impacted trust was linked to broader immigration policies and practices.

**Discussion:**

Findings underscore the need to expand trust models to include social drivers including policy and practice shaping Latino patients’ health care experiences. For example, non-health policies such as immigration policy contribute to care delays and impact health care trust. Understanding how trust is shaped by broader policy environments is essential to inform health systems that positively engage Latino populations.

**Supplementary Information:**

The online version contains supplementary material available at https://doi.org/10.1007/s11606-026-10591-4.

## INTRODUCTION

Trust, or the willingness to be vulnerable under conditions of risk and uncertainty, is critical to promoting timely entry and retention in health care and widely recognized as a pressing public health issue.^1^ Among marginalized communities in the United States (U.S.), trust in the health care system is low and often linked to experiences of discrimination.^2,3^ Trust in clinicians enhances access to care, improves quality, and leads to better health outcomes, including greater use of preventive services, medication adherence, and improved management of chronic conditions.^4,5^ In contrast, mistrust in health care institutions contributes to underuse of services, including missed appointments, delayed care, and unfilled prescriptions.^6,7^

Latinos are the fastest-growing population segment in the U.S. and Latino adults also report low levels of trust in their doctors.^8^ Despite this, most trust-related research has focused on other affected groups, such as Black and low-income patients.^4,9^ Even when Latino patients are included in studies, they are often underrepresented in research^10,11^ or studies fail to consider relevant social drivers that shape Latino patients’ health care experiences^12^ such as immigration history and documentation status. This is particularly concerning given that 35% of U.S. Latinos are foreign-born and many face exclusion from public health resources.^10,13^ To support just systems of care that promote health for all, we need insights into what shapes these low levels of trust among Latino patients. Without representative data and attention to relevant social contexts, clinical health care settings risk overlooking the needs of a population central to the nations’ demographic future.^14,15^

Scholars have shown that the use of integrated services like chronic disease management may decline without strong system support and trusting relationships.^5,16,17^ An estimated 12.6% of U.S. Latinos have diabetes, 27.1% have hypertension, and 33.5% have hyperlipidemia, all of which can contribute to complications, morbidity, and premature mortality.^18^ In trying to manage their chronic health conditions and maintain their health, Latino patients are simultaneously navigating social conditions, and systems and structures of care that shape their experiences and trust in engaging and accessing health care. Therefore, we must identify existing knowledge on trust in chronic disease management for these populations. This scoping review aimed to map key concepts related to trust among Latino patients in chronic care management in primary care, highlighting existing research and the experiences that shape trust in health care institutions.

## METHODS

A scoping review methodology was selected to systematically examine literature, identify research gaps, and clarify key concepts in this field.^19,20^ We conducted this scoping review in accordance with the Joanna Briggs Institute’s methodologic framework^20^ and followed the Preferred Reporting Items for Systematic Reviews and Meta-Analyses extension for Scoping Reviews (PRISMA-ScR) reporting guidelines.^21^ The review team collaborated with a research librarian M.H. to develop and execute a comprehensive literature search that combined controlled vocabulary and keyword terms related to trust/mistrust in Latinos and health care. A search was developed in PubMed and then translated into the following databases: Scopus (Elsevier), SocINDEX with Full Text (EBSCO), and APA PsycINFO (EBSCO) (see Appendix 1 for full search strategies). All searches were run between April 15 and 17, 2024, and limited to items published in English between the years of 2004 and April 17, 2024. For EBSCO-based databases, results were also limited to the source type of Academic Journals. Results were downloaded to a citation management software^22^ and then uploaded to a screening platform^23^ for deduplication.

### Study Selection

We established and applied consistent inclusion and exclusion criteria throughout study selection (see Appendix 2 for full criteria). Our search comprised only peer-reviewed studies, including systematic reviews, meta-analyses, and scoping reviews. Eligible studies focused on adults (18 +) in outpatient primary care management of chronic conditions. We excluded studies centered on specialty care, pediatrics, acute care, vaccine hesitancy, or medical research participation. OBGYN was considered primary care unless connected to hospital or surgical contexts. Mental health was included if managed within primary care; studies on specialty psychiatric care were excluded. To ensure data focused on Latino populations, we only included studies with at least 50% Latino participants, recognizing that studies with a significant number of Latino patients who did not meet this threshold may have been excluded. Additionally, we conducted a sensitivity analysis to identify potentially eligible multiethnic studies not captured within our original search strategy. This analysis yielded no additional studies meeting inclusion criteria. We use Latina/o/e/x and Hispanic interchangeably, using the U.S. Census Bureau definition of “Hispanic or Latino” as individuals who self-identify as being of Cuban, Mexican, Puerto Rican, South or Central American, or other Spanish culture or origin, regardless of race.^11,13^ We recognize concerns regarding different terms and varying preferences. We included studies published on or after January 1, 2004, to capture trends both before and after the 2010 Affordable Care Act and the effects of the COVID-19 pandemic. International studies were excluded due to differences in health care systems and approaches to care.

Using a two-person independent review process, the study team screened titles and abstracts for eligibility using the inclusion and exclusion criteria. Discrepancies were resolved through group discussion and consensus. Full-text screening followed the same process, with any conflicts again addressed collaboratively.

### Data Extraction

Data extraction was conducted for records meeting inclusion criteria after abstract and full-text screening and verified by a second team member. Data extraction took place between March 2025 and May 2025. We developed a custom data collection form in Covidence guided by the health systems trust content area framework, a conceptual framework derived from a systematic review of trust measures identifying eight key content areas of trust. ^10,24^ This form captured established trust domains (e.g., communication competency, fidelity, systems trust, confidentiality, fairness, stigma-based discrimination, global trust)^24^ and was further informed by the National Institute on Minority Health and Health Disparities (NIMHD) Research Framework^12^ to explore levels of influence relevant to care access (e.g., sociocultural environment, health care system characteristics), while allowing for identification of additional domains relevant to Latino patients.

Data extracted also included study characteristics, participant characteristics, chronic conditions, study results, and trust-related and non-trust-related utilization outcomes reported in each study. Operational definitions for each of these categories were based on their existing definitions.^12,24^ The data extraction form was piloted by the team prior to full extraction. Data extraction was conducted in Covidence, which flagged discrepancies for team review; all conflicts were resolved through group discussion and consensus.

### Data Synthesis and Analysis

We applied deductive content analysis, a method where codes are assigned to text or data based on an existing framework or theoretical structure derived from prior research and knowledge.^25^ In June 2025, the first and senior authors F.M.L and Y.A. conducted the analysis by first coding the data and then organizing codes into broader categories. If the data analyzed did not match the domains of the Health Systems Trust Content Area Framework or the NIHMD framework, we used inductive coding to code these additional observations and concepts to better reflect the unique health care experiences of these populations.^25^

## RESULTS

### Study Selection and Characteristics

**Figure 1:**
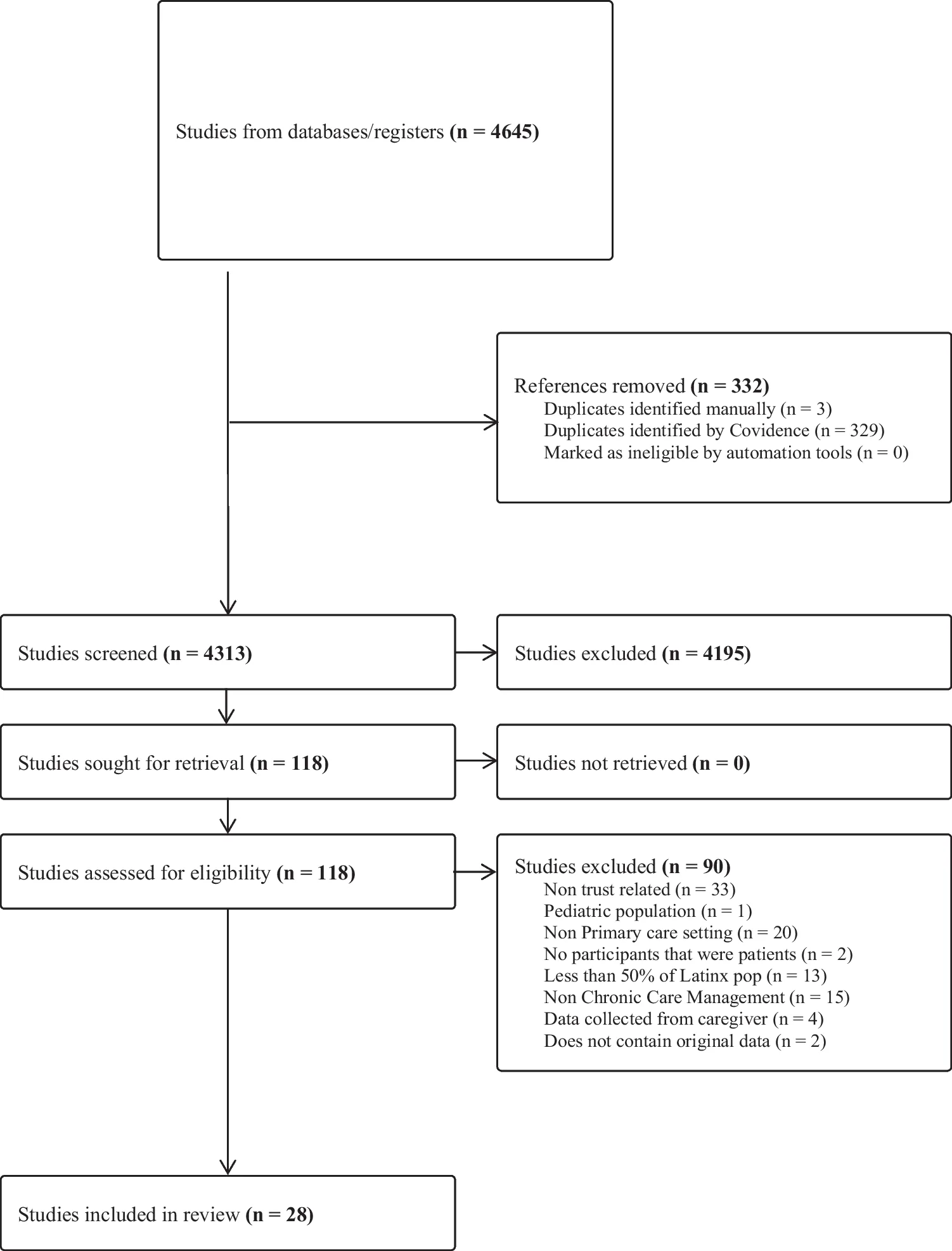
PRISMA flow diagram. Caption: The flow of study identification and selection.

The literature search retrieved 4645 records (Fig. 1). After removing duplicates, the study team screened the titles and abstracts of 4313 unique records for analysis eligibility. One hundred and eighteen full texts were reviewed, and twenty-eight studies met inclusion criteria.^14,15,26–51^ Descriptive characteristics are summarized in Table 1. Only three studies had a primary focus on trust or mistrust,^37,40,44^ while the remaining studies contained data referencing trust. More than half (53.5%) of the studies employed a qualitative design strategy.^15,26,28,31,32,36–40,45,46,48,49,51^ Studies encompassed a broad range of chronic medical conditions, with most of the studies focusing on diabetes.^26–29,31,33–37,39,43,44,47,49,51^

**Table 1:** Descriptives. Caption: Summary Characteristics of the 28 Studies

Characteristic	Studies, no
U.S.^14,15,26–51^	28
Study design	
Qualitative design^15,26,28,31,32,36–40,45,46,48,49,51^	15
Cross sectional study^27,41,43,50^	4
Mixed-method design^14,30,35,42,47^	5
Prospective observational study^29^	1
Randomized controlled trial^44^	1
Systematic review or scoping review^33,34^	2
Number of participants	
≤ 100^15,28,32,35–39,45,46,48,49,51^	13
101–500^14,26,27,29–31,40,43,44,47^	10
501–1000^41,42,50^	3
Not applicable^33,34^	2
Chronic medical conditions*	
Diabetes^26–29,31,33–37,39,43,44,47,49,51^	16
Hypertension^36,39,44,47,49^	5
Heart disease/CAD^35,43^	2
Hyperlipidemia^36,39,44^	3
Women’s health^40,42^	2
Chronic kidney disease^29^	1
Mental health^15,30,32,34,35,37,39,42,45–47,49^	12
Unspecified/other^14,27,31,34–36,38,41,43,48,50^	11

^*^The total count of chronic medical conditions exceeds 28 because several studies include populations with more than one condition

### Application of Trust Domain Categories

**Figure 2:**
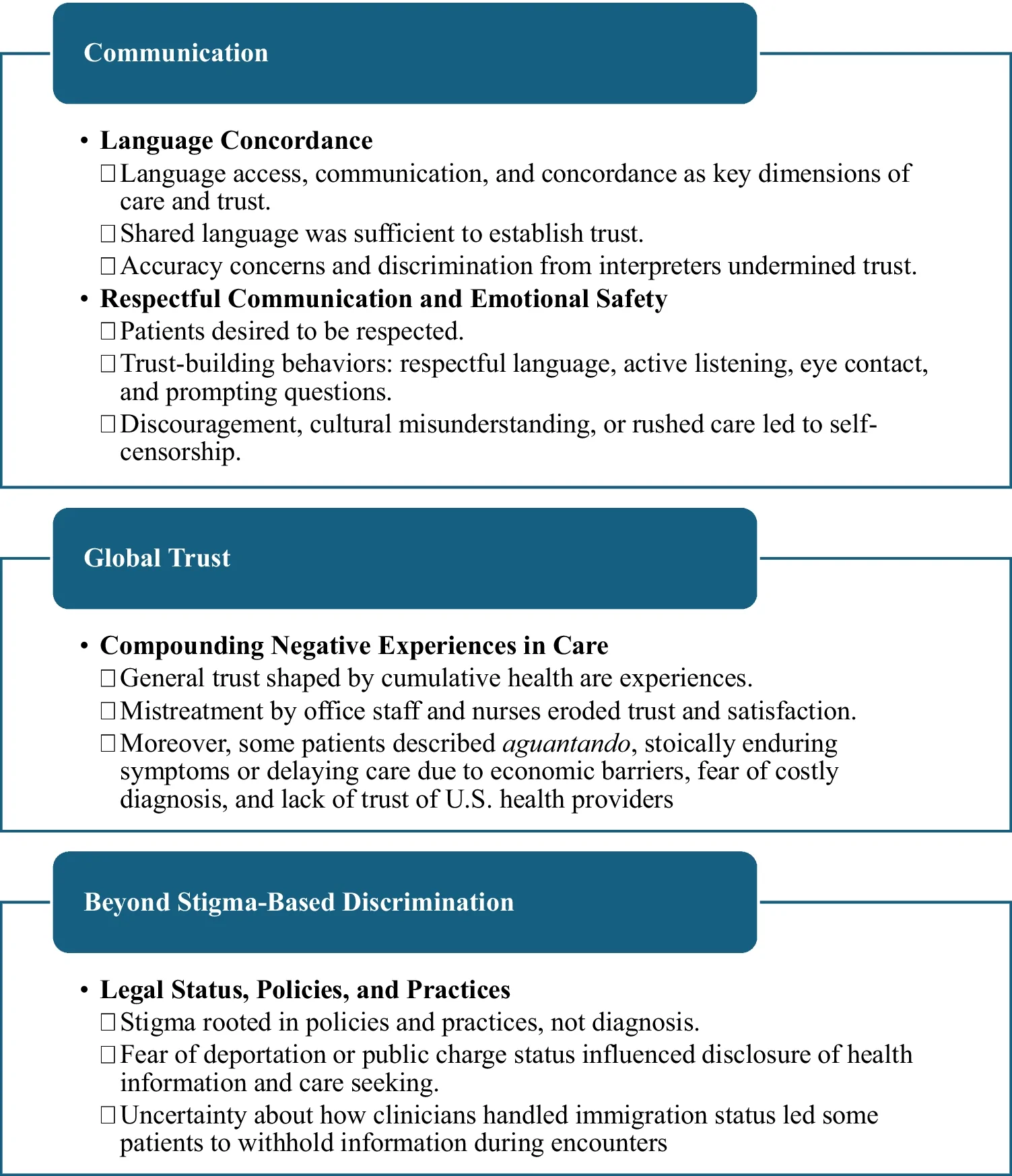
Summary of findings.

Using established trust domain categories, we first mapped each study accordingly and found most focused on communication (*N* = 20) and global trust (*N* = 23). Table 2 summarizes the distribution across all domains. Within these two domains, we identified dimensions reflecting Latino patients’ trust in health care settings that extend their existing definitions (Table 3). Findings also extended the stigma-based discrimination trust domain’s original focus beyond medical stigma (Fig. 2).

**Table 2:**
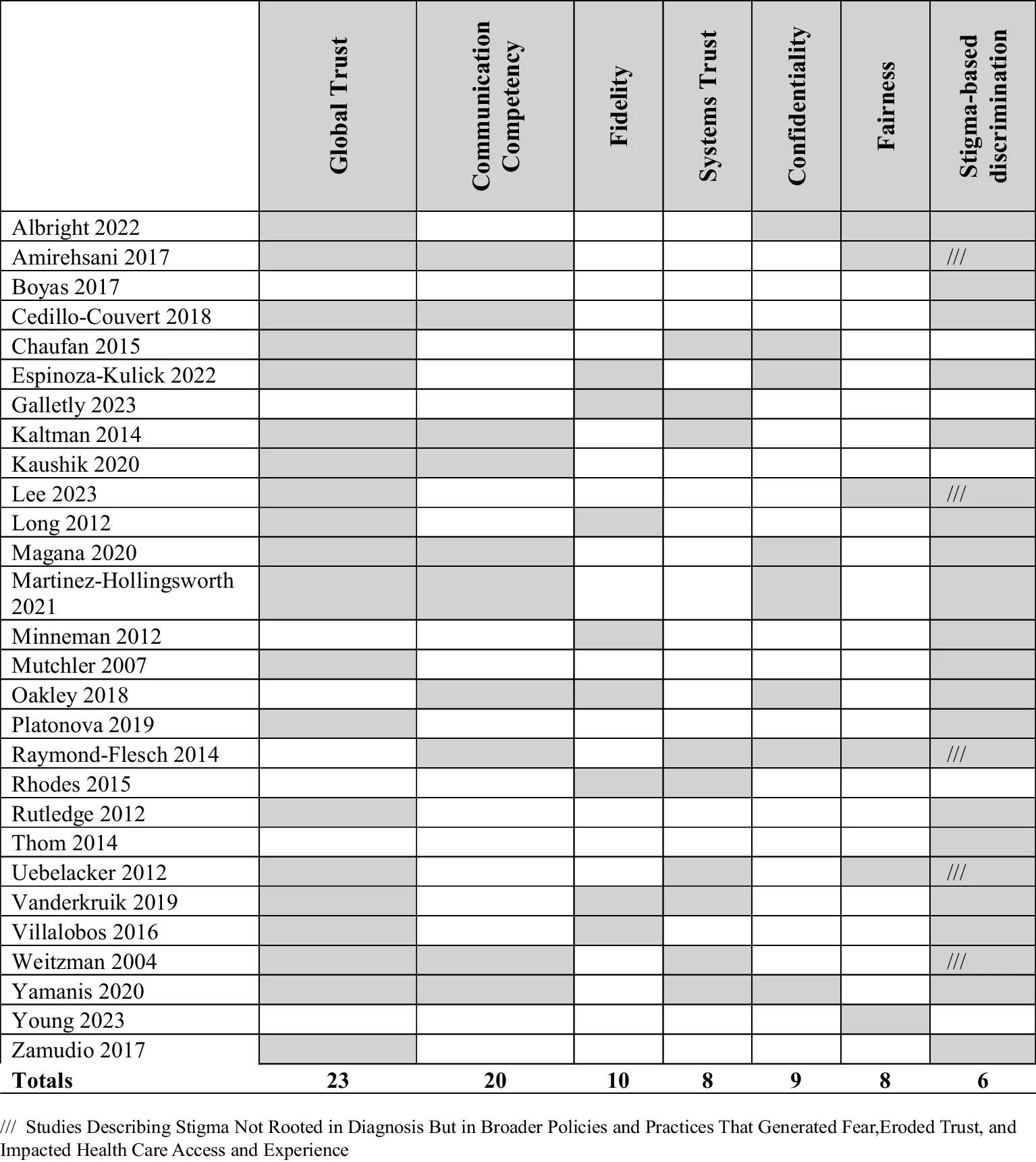
Distribution of Studies Across Trust Domains. Caption: Allocation of Studies Within Predefined Trust Domains

**Table 3:** Salient Trust Domains for Latino Patient Perspectives. Caption: Key Trust Domains Reflecting Latino Patients’ Trust and Examples from Studies

Studies	Examples
**Communication: language concordance**	
Kaltman (2014)^32^	“...What if the professional is not bilingual and has to look for someone to translate? It is not the same. The trust won’t be the same.”
Uebelacker (2012)^45^	“Yo a veces le he dicho al interprete: oye lo que tu estas diciendo el doctor no lo dijo” (“Sometimes I’ve told the interpreter: hey, what you’re saying, the doctor didn’t say that.”)
Mutchler (2007)^39^	“I felt very bad because … (the doctor) asked, ‘How long have you been here?’ I said, so many years. ‘Well, why can’t you speak English?’ Because I came here to work, to raise my family, and time slipped by. I didn’t know that was a sin. That’s what I told him.”
**Communication: respectful communication and emotional safety**	
Magaña (2020)^36^	“It depends on how they arrive, and greet you. Because sometimes they arrive very friendly, you feel comfortable when they treat you well, but when they arrive all like irritated, well no. That’s why I like going early because in the afternoon the doctors are already stressed, but there are some doctors that are kind and others that are very serious. If the doctor arrives very serious, then how can I trust him? I even forget what I was going to tell him because of seeing the way he is.”
Yamanis (2020)^49^	“I have stayed with him because he treats me well…He speaks a little bit of Spanish as well… Even though he speaks English and Spanish I trust him.”
Chaufan (2015)^28^	“Because if I had more trust…I would feel a lot more open to talk about how I feel… It is not that it is hard, or that I am shy, no. Quite simply, it is because the person I see has an interest, the person is interested, right? Then you feel more open. There is a much higher level of professionalism. So, you open up, you can talk about your life.”
Amirehsani (2017)^26^	“The nurses should be friendlier or the people working at the front desk when they provide information. They should be nicer because sometimes they are very rude and they say, “I already told you this. This is the way it is.” They don’t give you any other explanation”
**Global trust: compounding negative experiences in care**	
Weitzman (2004)^48^	“There are several doctors that simply tell you “what hurts?” Well, my finger hurts. “Well you will take these pills and that’s that.” They don’t try to talk with you. Sometimes your finger hurts because of other reasons... I have gone to some doctors that simply treat you in a doctor-patient way. And that is why you don’t trust them enough to tell them certain things.”
Martinez-Hollingsworth (2021)^37^	“I had printed out some studies and some articles. And I went in and I really wanted to discuss them with (my provider) … And I said, ‘Well, can we talk about this research that I did?’ She took the papers and threw them in the garbage and said, ‘That’s nonsense. Here’s your prescription. I’ll see you next year’”
Albright (2022)^14^	“… sometimes they discriminate against people because of their color, which has already happened to me … one of my daughters was discriminated against, because we went to a check-up that was teeth. I asked for the price and they said it was very expensive, not knowing that my daughter has medical coverage, just because they look at her brown color”
**Beyond stigma-based discrimination: legal status, policies, and practices**	
Weitzman (2004)^48^	“And if I’m dying of illness, I can’t go to the doctor. What if information [told to the doctor] is put in a computer? What if the people from immigration can see the information and see my address and come look for me.”
Galletly (2023)^31^	“…If you arrive with an advanced illness and you do not have documents, they will deny treatment and report you to be deported and you are going to die in your country, because this country does not want you to be a public burden.”
Raymond-Flesch (2014)^15^	“[doctors] don’t really know much about the status and what it means, necessarily, so sometimes the alternatives that they offer … don’t work for you because you’re not eligible for them.”'

#### Communication: Language Concordance

The established domain of Communication focuses on the quality, reliability, and nature of information exchange between all involved parties.^24^ However, existing definitions of this domain do not currently consider the role of shared language concordance, which is essential for achieving high-quality communication for this population. This review identified language access, communication, and concordance as key dimensions of patient care and trust-building.^34,39,47^ Spanish-speaking patients reported greater trust in clinicians who could discuss their health in their first language.^49^ Moreover, while some studies highlighted patient preference for clinicians with shared racial and cultural backgrounds,^45,46^ others found that shared language alone was sufficient to establish trust.^28,48,51^ In fact, bilingual clinicians were identified by patients as the most influential logistical factor in facilitating trust, with some patients indicating their willingness to pay more for language-concordant care.^32,46^

When clinicians did not speak Spanish, patients relied on interpreters, and limited access to these services was a barrier to trust.^14,30^ While interpreters facilitated communication, some patients felt involving an interpreter diminished the personal connection and trust they had with their clinician.^32^ Patients shared concerns about translation accuracy and reported instances where interpreters failed to fully convey their messages.^14,36,45^ Additionally, some patients experienced discrimination from interpreters, to which they attributed delays in receiving necessary, timely, or quality care. Despite these tensions, the absence of translation services altogether in a clinician’s office undermined trust with medical services broadly, particularly in settings associated with government institutions.^30^ In fact, challenges related to a lack of language access in patients’ care limited their willingness to take medical advice and ask follow-up questions during their visit.^33^

#### Communication: Respectful Communication and Emotional Safety

Beyond language access, communication competency was important for building trust. Communication competency emphasizes the technical and informational aspects of the patient and clinician encounter, such as listening, explaining treatment risks and benefits, and demonstrating clinical knowledge.^15,20^ Across studies, Latino patients desired to feel respected and engage in health conversations with their clinician in emotionally safe environments.^52,53^ In two studies, patients described a “trustworthy” clinician as someone whose behaviors included respectful language, active listening, eye contact, taking concerns seriously, and prompting questions to facilitate deeper conversations.^26,48^ Even during instances of language discordance, patients identified English-speaking clinicians as trustworthy due to continuity of care and their display of *simpatía*, a warm, caring demeanor that fosters harmonious relationships.^28,51^ Effective communication was further defined as clinicians taking a proactive approach in facilitating open communication through asking questions and creating space for the patient to ask questions.^26,28,41^ In contrast, when patients felt discouraged from asking questions, experienced cultural misunderstandings, or encountered doctors who were irritated or pressed for time, patients reported doubts about the quality of care, self-censorship to be respectful, or resorting to self-diagnosis or self-medication.^35,36,43,51^

#### Global Trust: Compounding Negative Experiences in Care

The global trust domain captures an individual’s overarching confidence in the trustworthiness of health care professionals and institutions. Several studies found direct associations between global trust metrics and health outcomes, showing that patients’ general trust was strongly shaped by their cumulative health care experiences.^27,29,37,40^ Patients reported that mistreatment or dehumanization by rude or dismissive office staff and nurses eroded their trust and satisfaction with care.^26,45,48^ While many expected support from health advocates, especially those of Latino descent,^30^ some felt these advocates made navigating the health care system more difficult.^48^

Additionally, patients’ repeated experiences of navigating high health care costs and the dissuasion of seeking care due to immigration status reduced opportunities to build trusting relationships and comfort with seeking health services.^15,49^ Patients described clinicians ordering excessive tests, rejecting outside clinic results, and charging for consultations without treatment, as examples of health care systems and clinicians prioritizing profit over care.^38^ Such experiences resulted in delays in care and perceptions of patients not receiving necessary, timely, and quality treatment.^42^ Moreover, some patients described *aguantando*, stoically enduring symptoms or postponing care in the hope the illness would self-resolve.^38^ Patients attributed *aguantando* to the lack of economic resources to seek medical care, fear of being diagnosed with an expensive illness, and the lack of trust in U.S. health providers, though additional characterization of this latter concept was not described.

#### Beyond Stigma-Based Discrimination: Legal Status, Policies, and Practices

The trust domain of Stigma-Based Discrimination examines how discriminatory treatment of patients with stigmatized conditions or behaviors (e.g., HIV and drug use) affects trust. In our analysis, Latino patients with chronic conditions experienced stigma rooted not in their diagnosis but in broader policies and practices, such as those conceptualized in the NIMHD framework.^12^ For Latino patients, policies and practices generated fear of deportation or uncertainty on eligibility for health care related to their immigration status that eroded trust and impacted health care access and experiences. For example, immigration policies and enforcement were perceived as contributing to stigma and prejudice.^30^ Fears of deportation enforcement or being labeled a public charge—a term used in U.S. immigration law when considering whether a person applying for legal permanent residency should be denied their legal status adjustment if they could become primarily dependent on the government for support in the future—influenced decisions on disclosing health information.^31,46^ Patients reported delaying care or avoiding driving, even in emergencies, to reduce the risk of detainment.^42^ Enforcement experiences were also associated with delays in care over the past 12 months.^50^ Moreover, the uncertainty with how clinicians handled their immigration status led some patients to withhold information during medical encounters.^15,45,48^ In fact, many patients sought clinicians they could trust not to expose them, considering medical encounters successful when they both received care and protected their legal status.^42,49^

## DISCUSSION

Few studies on trust in health care have centered on Latino populations,^4,9^ limiting our appreciation for issues relevant to the fastest-growing population segment in the U.S. To promote the health of all people, and support health care’s role in fostering trust that promotes timely access for its fastest-growing population, we set out to identify and describe the extent of research evidence at the intersection of trust and Latino identity. Analyzing 28 studies, we identified, mapped, and documented key concepts relating to Latino patients’ health care utilization trust within primary care. Notably, only three studies focused primarily on trust/mistrust, reiterating the gap in existing research focused on Latino perspectives. However, all included studies begin to tell the story of what influences trust for this understudied population.

Our findings suggest that while Latino patients’ experiences of trust in health care settings and of health care align with some established dimensions of trust, they are not fully reflected in existing conceptual models. Centering Latino patients’ perspectives using the Health Systems Trust Content Area Framework revealed the need to consider stigmatization of excludable groups within the organized structure of legal rights in the U.S. For instance, the stigma-based discrimination domain could extend to social drivers such as policies, practices, and experiences related to immigration and legal status,^12^ which significantly shaped trust in clinical care in this analysis and align with prior literature and levels of influence for care access defined in models for understanding health disparities, such as the NIMHD Research Framework.^54^

This study adds new insight into the nuanced role of interpreters in shaping Latino patients’ trust in health care. While research by Bazargan et al. has described communication as a central domain of trust for Latino patients,^3^ our findings reveal that interpreter use did not consistently foster trust. While access to interpreters is one solution to addressing language barriers among Latino populations, our findings that interpreter presence can diminish connection and trust suggest that health care team language capacity is necessary. Quality and accuracy of interpretation are also critical. Patients described concerns about interpretation accuracy, which led to miscommunication and emotional discomfort, eroding rather than building trust in these encounters. These experiences, especially when repeated across interactions with front desk staff, nurses, and interpreters, contributed to a gradual erosion of trust.^3,24^ This builds on existing scholarship demonstrating that respectful, language-concordant communication, marked by attentive listening, thoughtful questioning, and emotional safety, enhances trust.^55–58^ Conversely, the absence of these behaviors prompted patients to self-censor and question the quality of care. These findings underscore the importance of culturally and linguistically responsive communication across the entire care team for promoting trust and equity in health care delivery.

This analysis extends existing scholarship by contributing to our understanding on how policies and practices can hinder trust and care. Latino patients’ concerns about how their sensitive information might be used, particularly in relation to deportation, discrimination, and public charge rules, affected their willingness to engage, disclose, and seek care. Even in supportive and trusting clinical environments, these broader concerns contributed to the erosion of trust in institutions. These findings augment current conceptualizations of trust and care access and underscore that addressing such barriers is essential to fostering continued trust and improving access to care for Latino populations and for immigrant populations. Strengthening trust through primary care can enhance chronic disease management and continuity of care through the reduction of costly delays and service avoidance, thus improving health outcomes.^59^

This scoping review offers several strengths: first, centering Latino patients’ experiences, and doing so within the context of chronic disease management provides an important intersection in trust research. To help populations maintain health despite chronic conditions, it is necessary to understand facilitators and barriers to trust in care. Studies show that Latino patients utilize high-value preventive care;^17^ however, their use of more integrated services, like chronic care management, may decline without trusting relationships.^5^ Moreover, by comparing findings to existing trust domains,^10,24^ this study demonstrates where current models do not fully capture relevant dynamics for Latino populations. Unlike prior work focused on interpersonal factors, this review highlights the salience of contextual factors such as non-health policy and practices in shaping trust. Despite these strengths, several limitations remain. First, scoping reviews do not evaluate study quality. First, this study was not prospectively registered on a study registry prior to data collection. Second, this review was limited to peer-reviewed literature, which may have excluded relevant findings from nonprofit, government, and community-based organizations that study Latino patient experiences. Third, while the review focused on Latino patients, the existing research lacks an intersectional lens. Few studies disaggregated findings by other relevant social identities (e.g., gender, country of origin, length in the U.S. and immigration status) potentially obscuring important nuances in how trust is experienced across subgroups within this heterogenous population. This pattern reflects a broader gap in the field, and future research should prioritize intersectional approaches to examine within group diversity among Latino patients. However, literature indicates that irrespective of foreign-born/immigration status, Latino patients experience systems within the context of their identity, through objective characteristics (e.g., preferred language) and through narrative-based myths (e.g., undocumented immigrants overuse medical services) which spur differential treatment and experiences that could influence trust.^60^ Finally, although this review identified policies and practices as an emerging key concept, many included studies focused primarily on interpersonal trust rather than social drivers. Future studies should consider the range of social drivers and conditions that facilitate or impair trust and influence care-seeking and care-utilization among Latinos and in subpopulations experiencing stigmatized statuses in the U.S.’s structure of social belonging. Our analysis provides a foundation for future research and practice aimed at strengthening trust and improving health care experiences for Latino patients in the U.S.

## CONCLUSIONS

This scoping review makes visible an opportunity to study trust dimensions essential to understanding Latino patient experiences. Health care trust is shaped by interpersonal interactions and policy, which influence how Latino patients navigate health care. Clinical health systems must be cognizant of how these broader forces shape patients’ lives to respond accordingly to deliver equitable, patient-centered care.

## Supplementary Information

Below is the link to the electronic supplementary material.

Supplementary file1 (DOCX 18.3 KB)

## Data Availability

All data used in this scoping review were extracted from publicly published studies.
