# Supplementary material for: Dimensions of Trust in Health Care Experiences Among Latino Patients: A Scoping Review of the Literature
Source: J Gen Intern Med. 2026 Jul 7;41(14):4128–38. doi: 10.1007/s11606-026-10591-4 (PMC13728036; doi:10.1007/s11606-026-10591-4)
Supplement: Supplementary file 1 [file 11606_2026_10591_MOESM1_ESM.docx]

Appendix 1: Electronic Search Strategy

**PubMed**

(("Ambulatory Care"[Mesh] OR "Outpatient Clinics, Hospital"[Mesh] OR "Outpatients"[Mesh]) OR "Primary Health Care"[Mesh] OR "acute care"[tiab] OR "primary care"[tiab] OR ambulatory[tiab] OR outpatient[tiab] OR non-hospitalized[tiab])

AND

((("Trust"[Mesh] OR "Psychology, Social"[Mesh] OR "Avoidance Learning"[Mesh] OR "Conditioning, Psychological"[Majr] OR "Conditioning, Operant"[Mesh] OR "Attitude to Health"[Mesh] OR "Patient Acceptance of Health Care"[Mesh] OR trust[tiab] OR distrust[tiab] OR mistrust[tiab] OR attitude[tiab] OR "belief system"[tiab] OR psychological[tiab] OR conditioning[tiab] OR disparit*[tiab] OR barriers[tiab] OR experiences[tiab] OR perspectives[tiab] OR experiences[tiab] OR "psychosocial factors"[tiab]))

AND

("treatment avoidance"[tiab] OR "health seeking"[tiab] OR accessibility[tiab] OR "barriers to health"[tiab] OR "delayed care" OR "delay in treatment" OR "health care" OR "health service"[tiab] OR "health behavior" OR "Health Services Accessibility"[Mesh] OR "medical care" OR "Physician-Patient Relations"[Mesh] OR "help seek*")

AND

("Hispanic or Latino"[Mesh] OR "Minority Groups"[Mesh] OR Latino[tiab] OR Latina[tiab] OR Latinx[tiab] OR Hispanic*[tiab] OR "Spanish speaking"[tiab] OR "Emigrants and Immigrants"[Mesh] OR "Undocumented Immigrants"[Mesh] OR immigrant*[tiab])

NOT

("African American"[tiab] OR Black[tiab] OR non-Hispanic[tiab] OR White[tiab] OR Canada OR "UK" OR "NHS" OR "United Kingdom" OR Korean OR Africa))

Limits: 2004-2024, English

**PsycINFO**

DE "Trust (Social Behavior)" OR TI ( trust or trusting or trustworthiness or trustworthy OR mistrust OR distrust or abuse or racism ) OR AB ( trust or trusting or trustworthiness or trustworthy OR mistrust OR distrust or abuse or racism )

AND

DE "Latinos/Latinas" OR DE "Mexican Americans" OR DE "Central American Cultural Groups" OR DE "South American Cultural Groups" OR TI ( latina OR latino OR latinx OR hispanic* OR "Spanish speaking" OR "immigrant patients" OR immigrant )

AND

( ((DE "Health Care Access" OR DE "Health Care Services" OR DE "Health Care Utilization" OR DE "Health Disparities") OR (DE "Treatment Barriers")) OR (DE "Treatment Refusal") ) OR TI ( health care or healthcare or disparit* or health services or health access or medical care OR "delayed diagnosis" OR "delay in care" OR "delayed care" OR "health seek*" or "treatment avoidan*" OR “help seek*” ) OR AB ( health care or healthcare or disparit* or health services or health access or medical care OR "delayed diagnosis" OR "delay in care" OR "delayed care" OR "health seek*" or "treatment avoidan*" OR “help seek*”)

AND

(TI ( experiences or perceptions or attitudes or views or feelings or qualitative or perspective or utilization OR utilizing OR using ) OR AB ( experiences or perceptions or attitudes or views or feelings or qualitative or perspective or utilization OR utilizing OR using ))

Limits engaged: 2004-2024, Academic journals

**SocINDEX**

*(((DE "TRUST" OR DE "SUSPICION" OR DE "ATTITUDE (Psychology)" OR DE "MEDICAL mistrust")) OR DE "EXPERIENCE" OR trust OR mistrust OR distrust OR suspicion OR experienc* OR attitude)*

*AND*

*((DE "MEDICAL care" OR DE "HEALTH services accessibility" OR DE "MEDICAL care of ethnic groups" OR DE "MEDICAL care of immigrants" OR DE "MEDICAL care of minorities" OR DE "PRIMARY health care" OR DE "OUTPATIENT medical care" ) OR TI ( primary care or health care or healthcare or medical care or ambulatory or outpatient or treatment barrier* OR barrier to treatment OR delay in care OR utilization OR access OR health-seeking OR help seeking)*

*AND*
*( (DE "HISPANIC Americans" OR DE "RACIAL & ethnic attitudes") OR (DE "MINORITIES") ) OR TI ( spanish speak* or latino or Latina or latinx or hispanic* or minorit* or immigrant* OR central american or south american )*

Limits engaged: 2004-2024, Academic journals

**SCOPUS**

( TITLE ( latin* OR hispanic* OR {Spanish speaking} ) AND TITLE-ABS-KEY ( trust* OR mistrust OR distrust OR belief OR fear OR acceptance OR {social conditioning} OR disparit* OR abuse OR racism ) AND TITLE-ABS-KEY ( {medical care} OR {health seek*} OR {help seek*} OR {health care} OR {delay W/3 care} OR {treatment avoidan*} ) ) AND PUBYEAR > 2003 AND PUBYEAR < 2025 AND ( LIMIT-TO ( DOCTYPE , "re" ) OR LIMIT-TO ( DOCTYPE , "ar" ) )

**SENSITIVITY ANALYSIS**

We re-ran our search removing the following NOT clause to identify potentially eligible multiethnic studies not captured by our original search strategy.

NOT

("African American"[tiab] OR Black[tiab] OR non-Hispanic[tiab] OR White[tiab] OR Canada OR "UK" OR "NHS" OR "United Kingdom" OR Korean OR Africa))

Appendix 2: Inclusion and Exclusion Criteria

| **Inclusion criteria** | **Exclusion criteria** |
| --- | --- |
| Trust in doctors, health care teams, health care institutions, health care as an institution, inclusive of extensions of the health care system structure such as Community Health Workers (CHW’s) |  |
| Medical mistrust, distrust, trustworthiness |  |
| Adult populations (>18yo) | Pediatric population |
| Outpatient primary care setting including: Family Medicine, Internal Medicine, Obstetrics and Gynecology | Acute care, Inpatient/Hospital setting, Specialty care including substance use/abuse, hospice/palliative/end-of-life care, cancer care |
| Chronic care management: diabetes, hypertension, hyperlipidemia, mental health | Preventive screening |
| Trust and: Health care access, delays in care, adherence, non-adherence, avoiding care, utilization | Trust and: vaccines, vaccine uptake, participation in research about vaccines |
| Trust and: Health outcomes | Trust and: research participation |
| Within the US | Outside of the US or Canada |
| Hispanic/Latino: Latin American background. Including Immigrant and US born |  |
| >50% of the research subjects are Latino |  |
| Peer reviewed | Dissertations |
| Contains original data: Prospective, retrospective, qualitative studies, observational studies, secondary analyses, clinical trials | Protocol Papers, Editorials, or Commentaries |
| Systematic Reviews, Meta-Analysis, Scoping Reviews |  |
| Published on or after January 1, 2004 |  |
